# Hospitalization and help-seeking among first episode psychosis patients

**DOI:** 10.1007/s44192-024-00064-7

**Published:** 2024-04-03

**Authors:** Anna Yee, Sarah Greene, Ashley Weiss, Serena Chaudhry, Spencer Steadman

**Affiliations:** 1 School of Medicine, Tulane University, New Orleans, LA USA; 2 Providence Sacred Heart Medical Center, Spokane, WA USA

**Keywords:** First-episode psychosis, Coordinated specialty care, Hospitalization, Duration of untreated psychosis, Help-seeking episodes

## Abstract

**Purpose:**

To examine hospitalization as part of a complex pathway to care in first episode psychosis (FEP), exploring help-seeking episodes (HSE) and their relationship to hospitalization.

**Methods:**

Data from 66 patients at the Early Psychosis Intervention Clinic New Orleans (EPIC-NOLA), a coordinated specialty care (CSC) clinic, was obtained from Pathways to Care (PTC) assessments, which documents elements of help seeking. A chart review was performed identifying hospitalizations.

**Results:**

Most patients were hospitalized multiple times (n = 37, M = 2.98, SD = 2.14). On average, patients had more hospitalizations prior to starting treatment at EPIC-NOLA (M = 1.72, SD = 1.35) than after (M = 1.27, SD = 1.79). Patients whose first HSE resulted in intake at EPIC-NOLA were significantly less likely to be hospitalized after intake than patients with multiple HSE (*F*(1,52.3) = 12.9, p < .001). There was a significant correlation (N = 42) between HSE and hospitalizations after intake (τb = .327 p < .05); patients seeking help more often were more likely to be hospitalized after intake. No significant correlations were found between duration of untreated psychosis (DUP) and hospitalization.

**Conclusion:**

While results are correlational, several key relationships were noted. Fewer hospitalizations occurred after intake into EPIC-NOLA. Starting treatment after the first HSE was related to fewer future hospitalizations, compared to intake after multiple HSEs. Intake into a CSC clinic after a single HSE may reduce hospitalization. Additionally, increased HSE, not DUP, impacted patients' likelihood of hospitalization. This prompts treatment engagement during a first HSE to reduce hospitalization.

## Introduction

The early recognition of psychosis due to underlying psychotic disorders is critical, as these conditions may have debilitating sequelae and cause early mortality [1]. Coordinated Specialty Care (CSC) at Early Intervention Clinics (EIC) plays a potentially important role in early recognition and treatment of first episode psychosis (FEP), with a goal to decrease the duration of untreated psychosis (DUP) to improve outcomes for patients [2–4]. A systematic review found that team-based early intervention services such as the CSC model are superior to regular care across multiple outcome measures, allowing for improvement in symptom severity, enhanced quality of life, lower relapse rates, and lower risk of psychiatric hospitalizations [5].

Focus on care models that improve outcomes is valuable because recovery after first episode psychosis is generally poor. One study on early psychosis treatment found only 13.7% of their subjects met criteria for full recovery after 2 years, defining recovery as remission of positive and negative symptoms and adequate social/vocational functioning [6]. Another meta-analysis found that only 1 in 7 patients with schizophrenia achieve recovery [7]. One contributing factor to these poor treatment outcomes is a long duration of untreated psychosis (DUP) [8]. A meta-analysis exploring DUP as a long-term predictor of outcomes found reducing DUP decreased severe positive and negative symptoms, increased likelihood of remission, and improved social functioning [9]. One large systematic review estimated that the average DUP exceeds 100 weeks [10]; a more recent trial in the United States found the median DUP to be 74 weeks [2]. The excessive length of time between first psychotic symptoms and intake to specialist care for these symptoms may be explained by many factors, such as lack of awareness regarding psychosis and treatment resources, stigma, or the belief that they can manage their symptoms without treatment [11, 12].

During this time of untreated psychosis, a common problem is increased risk for hospitalization [13, 14]. Predictors for hospitalization in this population include prior hospitalizations, longer DUP, substance use, presence of positive symptoms, and belief about the value of medication [8, 15, 16]. Hospitalizations prior to starting treatment at an early intervention program are a significant predictor of hospitalization after starting treatment [17]. Some studies found shorter DUPs were associated with less hospitalizations prior to intake and a reduction in re-hospitalizations [17, 18], although a meta-analysis reports overall no correlation between DUP and hospital stays [9]. While both longer DUP and hospitalizations have been associated with poorer outcomes in FEP care, there is a lack of research evaluating these in the context of the individual’s unique pathway to care and the associated help seeking episodes (HSE). This study aims to explore this relationship.

In this study, HSEs for each patient were quantified as any moment that a patient’s psychotic symptoms led them to seek and/or receive support or treatment. This includes confiding in others, seeking or being referred to resources or professional services, and hospitalization. A prior study among this clinic population found that patients who experienced only hospitalizations while seeking help had significantly longer DUPs than those who sought help from more than just hospitals, for instance family and self-referrals [19]. This suggested that individuals with a mix of help seeking behaviors involving the individual, family, and hospitals had significantly shorter DUPs than people only help-seeking at a hospital. Identifying these pathways with shorter DUPs is critical, given shorter DUP is associated with improved quality of life and symptoms over time [2]. This illuminated the need for further analysis on how outcomes like DUP and hospitalizations are potentially influenced by one's help-seeking journey, and what specific help seeking patterns in a patient’s pathway to care can increase positive outcomes.

This study was conducted at the Early Psychosis Intervention Clinic New Orleans (EPIC-NOLA). EPIC-NOLA is a CSC clinic providing FEP services to adolescents and young adults experiencing FEP. The study sample catchment area includes the greater New Orleans area, but due to the dearth of services across Louisiana, EPIC-NOLA occasionally provides services to patients from other parishes and regions of Louisiana. The clinic provides services to primarily 12–35 year olds, and patients are accepted regardless of insurance status or type. EPIC assists uninsured patients in applying for Medicaid in order to pay for services. In the meantime, these uninsured patients can utilize the Mental Health Block Grant to cover their treatment costs at EPIC-NOLA.

We will evaluate the following hypotheses:

### *Hypothesis 1*

Patients whose first HSE resulted in a referral and treatment at EPIC-NOLA have less hospitalizations after intake, compared to those with 2 + HSE.

### *Hypothesis 2*

Shorter DUPs are associated with less hospitalizations after intake.

## Methods

This is a retrospective chart review study on a sample of convenience. The sample was obtained from active patients at EPIC-NOLA, a CSC clinic providing care for FEP patients. Patients were contacted after a regular clinic appointment to complete a Pathways to Care (PTC) assessment, a semi-structured assessment which details a patient's psychotic-symptom-related encounters with the community prior to admission to our clinic. The PTC assessment organizes these encounters into help seeking episodes (HSE). A HSE describes any time a patient or community member sought help for symptoms of psychosis. The HSE includes when help-seeking occurred, who initiated, why it occurred, and the outcome of the help seeking. This does not include help-seeking for prodromal symptoms, but starts at acute onset of psychotic symptoms. While there are not currently validated tools for studying pathways to care, prior studies assessing patient pathways support looking at both empiric service utilization and dynamic interpersonal factors [26]. The PTC Assessment used for this study captures both service utilization (documenting contact with healthcare services) and personal factors (documenting who and why help seeking was initiated), therefore is appropriate for this study.

DUP is calculated as the time between when a patient first noticed symptoms of psychosis to intake at EPIC-NOLA. In the literature, there are variations in the definition of DUP: some start DUP at onset of prodromal symptoms, or stop at time of first anti-psychotic medication. We do not model these different parameters in this study. While prodromal symptoms and first medication are important factors in early psychosis treatment, this study focuses on patients' arrival and treatment at a CSC clinic. Thus using a DUP based on acute psychotic symptoms and treatment starting at the CSC clinic is the ideal way to define DUP for this study.

To determine a patient’s history of hospitalizations, a chart review was conducted of patients who completed PTC. All notes of our patient sample were reviewed in the EPIC-NOLA EMR (eClinicalWorks) using a key word search of “hospital” and “inpatient.” All documented hospitalizations from notes dating November 2018-May 2022 were included (this includes any hospitalization documented, including hospitalizations prior to this date range). Inclusion criteria included: 18 years of age or older, having completed a PTC assessment, and clear documentation regarding hospitalizations in their eClinicalWorks chart.

## Patients

Sixty-six patients met inclusion criteria. This patient group (Table 1) was mostly male (n = 48) and Black (n = 37) with schizophrenia as the most common diagnosis (n = 28). Referrals most commonly came from inpatient stays (n = 37). Twenty-two patients (33%) were referred to EPIC-NOLA after their first help-seeking episode. Medicaid was the most common insurance (n = 47).

**Table 1:** Demographics

	Counts	% of Total
**Gender**		
Male	48	72.7%
Female	18	27.3%
**Race**		
Black	37	56.1%
White	22	33.3%
Hispanic	4	6.1%
Unknown	2	3.0%
Asian	1	1.5%
**Diagnosis**		
Schizophrenia	28	42.4%
Unspecified psychosis	13	19.7%
Bipolar I	10	15.2%
Schizoaffective	9	13.6%
Major Depressive Disorder w/ psychotic features	2	3.0%
Schizophreniform	2	3.0%
Substance Induced Psychotic Disorder	1	1.5%
Narcolepsy	1	1.5%
**Insurance**		
Medicaid	47	71.2%
Community	18	27.3%
No Insurance	1	1.5%
**Referral source**		
Inpatient	37	56.1%
Self	13	19.7%
Outpatient	9	13.6%
Family	4	6.1%
Juvenile justice	2	3.0%
Emergency department	1	1.5%

## Results

Of 66 patients, 61 reported being hospitalized at any point in time for psychosis symptoms, only 5 patients had never experienced any hospitalization related to psychosis. Fifty-five patients experienced hospitalization before intake, 36 patients experienced hospitalization after intake. The mean number of hospitalizations was 2.98 (SD = 2.14); most patients were hospitalized more than once (n = 38). On average, patients had a greater number of hospitalizations prior to starting treatment at EPIC-NOLA (M = 1.72, SD = 1.35) than after (M = 1.27, SD = 1.79). The mean number of HSE was 2.13 (SD = 1.23).

For this study, the independent variable is the number of HSE, as documented by the PTC assessment. The dependent variables are DUP, and hospitalizations (broken down into subcategories of hospitalization before intake, after intake, and total hospitalizations). Correlations were evaluated with the Kendall rank correlation coefficient and the significance threshold was set at 0.05.

**Fig. 1:**
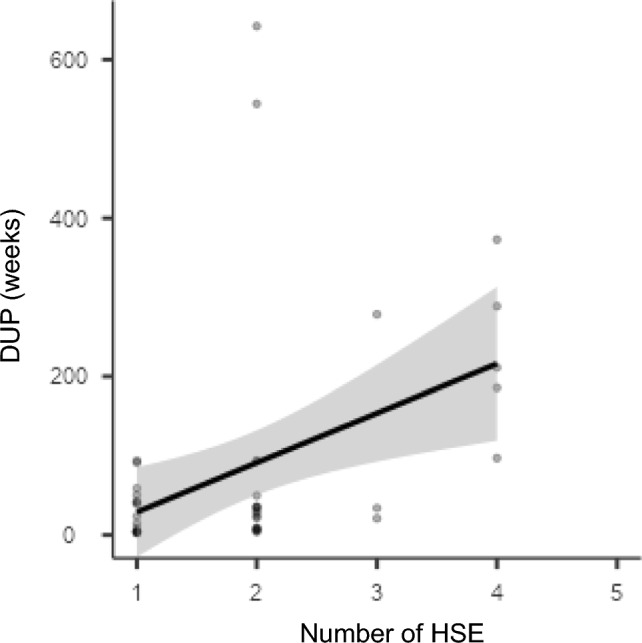
Correlation between HSE and DUP

HSE and DUP were positively correlated (N = 42 τb = 0.34 p < 0.05) (Fig. 1).

Both hypotheses were evaluated:

### Hypothesis 1

Patients whose first HSE resulted in a referral and treatment at EPIC-NOLA have lower rates of hospitalization after intake, compared to those with 2 + HSE.

**Fig. 2:**
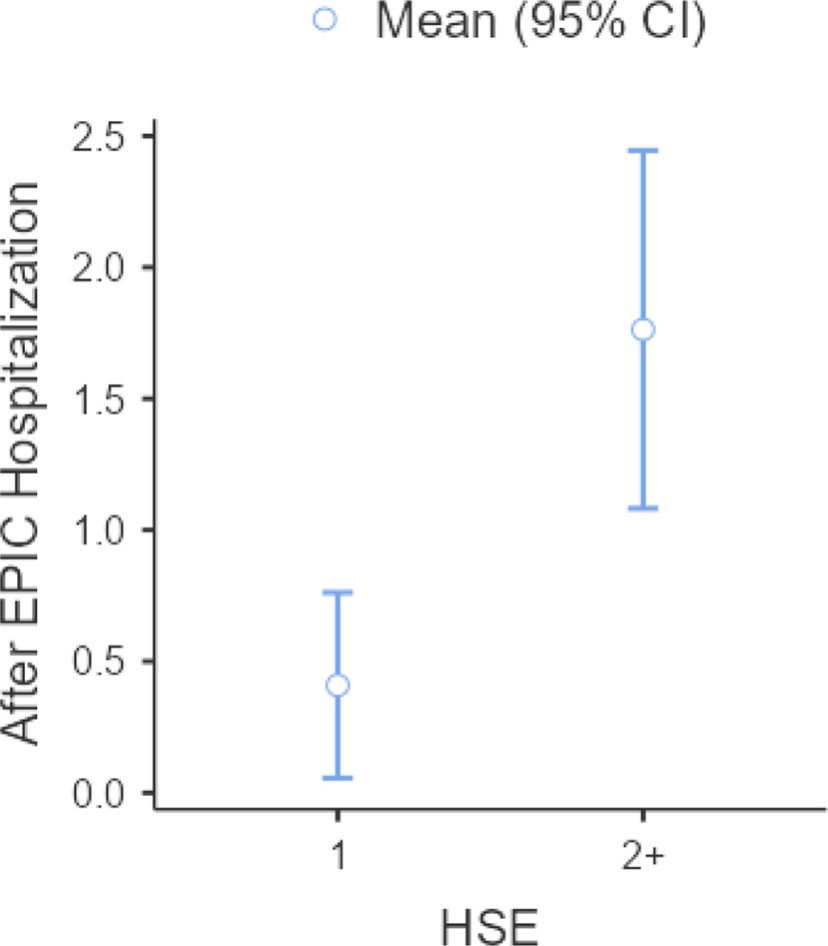
One HSE vs 2 + HSE and Number of Hospitalizations after intake at EPIC-NOLA

Hypothesis 1 was supported. A one-way ANOVA demonstrated patients whose first HSE resulted in intake at EPIC-NOLA were less likely to be hospitalized after intake than patients with multiple HSE; this result was statistically significant (*F*(1,52.3) = 12.9, p < 0.001) (Fig. 2)(Table 2).

**Table 2:** Table for Fig. 2

	HSE	N	Mean	SD	SE
After EPIC hospitalization	1	22	0.409	0.796	0.170
	2 +	38	1.763	2.072	0.336

**Fig. 3:**
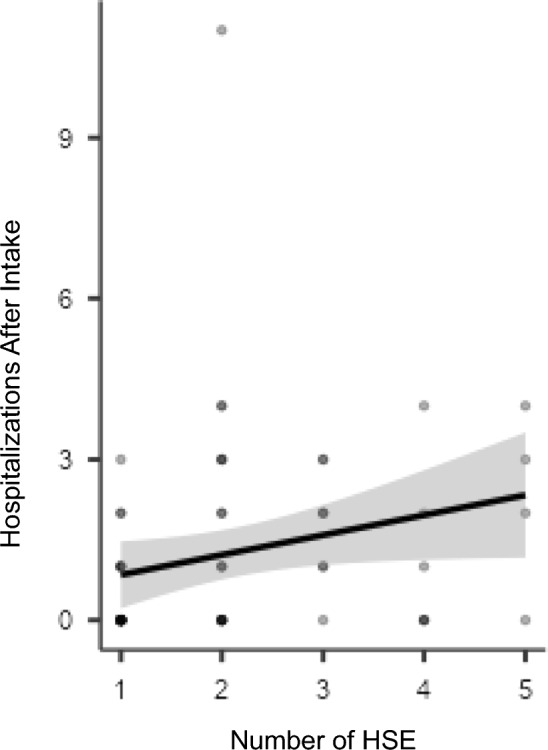
Correlation between HSE and Hospitalizations after Intake

Additionally, there was found to be a significant correlation (N = 60) between number of HSE and hospitalizations after intake (τb = 0.327 p < 0.05) (Fig. 3).

### Hypothesis 2

Shorter DUPs are associated with less hospitalizations after intake.

Hypothesis 2 was not supported. No significant correlations were found between DUP and hospitalization.

## Discussion

The goals of this study were to examine patterns of help seeking to understand how they impact hospitalization outcomes in FEP. This study showed hospitalization is extremely common in the early course of psychosis for our patients. One unique finding that emerged was a positive correlation between HSE and after-intake hospitalizations. This shows that patients who seek help more often prior to entering treatment at EPIC-NOLA, are hospitalized more after intake at EPIC-NOLA. This is a unique finding that, to our knowledge, has not been explored in the literature. Perhaps it is related to a lower threshold to seek help at hospital services, or stronger support networks to initiate help-seeking. This could represent a global increase in utilization of services as it is related to help seeking–if patients are more willing to seek help at baseline, this trait could be present during the onset of distressing symptoms as well as after starting treatment. This result could be confounded by greater severity of illness, as perhaps more help seeking could indicate greater symptom severity, and thus more hospitalizations throughout their disease course. Further research is needed to investigate this relationship. Ideally, early detection campaigns can help alleviate this, with the goal to perpetuate direct patient help seeking to CSCs. Consequently, patients may experience fewer HSE and fewer hospitalizations.

Our results also support that CSC at EPIC-NOLA can impact hospitalization, as hospitalizations decreased after initiation of treatment. It is unknown whether this is due to the specialty care of an early intervention clinic or simply entering care sooner, as our study design only looks at patients within an FEP clinic without comparing to general outpatient psychiatric treatment. Regardless, this supports that quick intake into outpatient treatment, specifically with CSC, may be beneficial in FEP to reduce hospitalizations experienced.

While this sample overall had reduced hospitalizations after starting treatment, we wanted to see if the quantity of help-seeking was a factor within the group. This led to a key finding that patients with 1 HSE before intake at EPIC-NOLA had fewer after-intake hospitalizations, compared to those with multiple HSEs. Essentially, patients ending up at EPIC-NOLA after the first time they seek help are less likely to be hospitalized in the future than those patients who sought help multiple times. This suggests increased focus should be placed on helping patients access CSC programs after their first time seeking help to reduce detrimental outcomes like repeated hospitalizations. One way in which CSC clinics can achieve earlier presentations in the FEP patient population is through developing programs that focus on identifying these help-seeking patterns. For instance, EPIC-NOLA has developed a parallel psychosis early detection program, CALM-Clear Answers to Louisiana Mental Health. CALM is modeled after the pioneer psychosis early detection campaigns, TIPS [20, 21] and MindMap [22]. CALM’s strategy and activity is informed by outcomes such as the ones presented in this paper. CALM involves the community, family members, and medical systems in properly identifying and referring to outpatient treatment for first episode psychosis, before hospitalization. It is possible that CALM’s efforts to educate the community on seeking the right help for FEP in New Orleans could have contributed to these findings at EPIC-NOLA.

One surprising finding was no relationship between shorter DUPs and after-intake hospitalization at EPIC-NOLA. Prior studies have variable findings on whether DUP impacts hospitalization [9, 17, 18], yet we expected shorter DUPs to be associated with fewer hospitalizations given shorter DUPs generally improve outcomes. This may demonstrate that hospitalization is an outcome less related to DUP than previously expected. It may be beneficial for future analysis to examine DUP and hospitalization in the context of the help-seeking journey. Focusing on hospitalization does not entirely reflect a patient’s wellbeing and individual experiences; using hospitalization as an outcome can place increased emphasis on services rather than patient wellbeing. Therefore, focusing on individual-specific help seeking could be valuable, and may be more strongly related to hospitalization than just DUP.

Ultimately, focus on the early pre-treatment period is critical. It is a discouraging reality, as found in this study and many others, that patients with FEP experience multiple HSEs without treatment and long DUPs complicated by frequent hospitalizations. These are clearly identified issues in the process of providing care for FEP patients. A reduction in hospitalizations, or even elimination of hospitalization as an expected part of the FEP help seeking journey, is aspirational for several reasons. Rehospitalization among the first episode psychosis population carries a high burden of direct healthcare costs [23]. Additionally, hospitalization can be a disruptive and traumatizing experience for patients and their families [24] which is consistent with the finding that patients endorse reduced hospitalizations as a clinically-based treatment goal [25]. Supporting efforts to target initial HSEs to decrease DUP and hospitalizations could have widespread benefits to this patient population.

## Limitations

Limitations to this study include sample size, limitations of the assessment tool, and the retrospective chart review. Our small sample size limits statistical power and generalizability. The primary measure of this study (Pathways to Care Assessment) may also have limitations. While it is a structured assessment, it relies on self-report from patients during a vulnerable period of recovery, leading to subjective variability. Patients may not accurately remember the number of times they sought help and may be biased towards memories of help seeking that involved impactful events like hospitalization, rather than a less impactful help seeking like talking to a friend or family member. Additionally, these assessments were completed with patients at various times after initiating treatment. Some patients completed it within weeks of intake, others after years. This limits the subject’s ability to accurately recall due to the passage of time, and could impact the reliability of this tool. Another limitation is the subjective reporting of hospitalizations prior to intake in the chart review. Hospitalization prior to intake was reported by patients to providers during initial intake, therefore is susceptible to poor recall of past events. However, given the multiple sources of information including records review, collateral sources, patient report, and treatment team case formulations, efforts were made to minimize this. Additionally, most of our findings are consistent with prior literature and support common findings in FEP research. Further exploration of DUP, HSE, and hospitalization should be conducted to identify ways to decrease the burden of hospitalization on these patients.

## Conclusion

People experiencing FEP are likely to face hospitalization during their illness. Reducing hospitalizations for these patients is a primary focus of CSC clinics to decrease healthcare cost burden, prevent emotional disruption, and shorten DUP to improve outcomes. Our results show that hospitalizations decrease after initiation of treatment at EPIC-NOLA. They also suggest patients whose initial help-seeking led them to EPIC-NOLA have lower hospitalizations after starting treatment, supporting the potentially pivotal role of community engagement strategies along with CSC clinics in reducing hospitalization. While this study did not report any relationship between DUP and hospitalization at EPIC-NOLA, perhaps this indicates a more individualized approach–focusing on the amount and type of HSE–is more valuable in reducing hospitalizations than simply focusing on DUP. Our final finding showed increased help-seeking was correlated with increased after-intake hospitalizations, a new finding which needs further investigation to identify causal relationships. Ultimately our goal is to reduce–or even eliminate–hospitalizations for people experiencing psychosis, finding ways in which patients can receive outpatient treatment with CSC as early in the disease course as possible to live fulfilling lives without disruption.

## Data Availability

The data that support the findings of this study are not openly available to preserve individual privacy and are available from the corresponding author upon request. Data are stored in a secured Redcap database affiliated with Tulane University and the Early Psychosis Intervention Clinic of New Orleans.
